# antiSMASH 8.0: extended gene cluster detection capabilities and analyses of chemistry, enzymology, and regulation

**DOI:** 10.1093/nar/gkaf334

**Published:** 2025-04-25

**Authors:** Kai Blin, Simon Shaw, Lisa Vader, Judit Szenei, Zachary L Reitz, Hannah E Augustijn, José D D Cediel-Becerra, Valérie de Crécy-Lagard, Robert A Koetsier, Sam E Williams, Pablo Cruz-Morales, Sopida Wongwas, Alejandro E Segurado Luchsinger, Friederike Biermann, Aleksandra Korenskaia, Mitja M Zdouc, David Meijer, Barbara R Terlouw, Justin J J van der Hooft, Nadine Ziemert, Eric J N Helfrich, Joleen Masschelein, Christophe Corre, Marc G Chevrette, Gilles P van Wezel, Marnix H Medema, Tilmann Weber

**Affiliations:** The Novo Nordisk Foundation Center for Biosustainability, Technical University of Denmark, 2950 Kongens Lyngby, Denmark; The Novo Nordisk Foundation Center for Biosustainability, Technical University of Denmark, 2950 Kongens Lyngby, Denmark; The Novo Nordisk Foundation Center for Biosustainability, Technical University of Denmark, 2950 Kongens Lyngby, Denmark; The Novo Nordisk Foundation Center for Biosustainability, Technical University of Denmark, 2950 Kongens Lyngby, Denmark; Department of Ecology, Evolution and Marine Biology, University of California, 1169 Biological Sciences II, Santa Barbara, CA 93106, United States; Institute of Biology, Leiden University, Sylviusweg 72, 2333 BE Leiden, the Netherlands; Bioinformatics Group, Wageningen University & Research, Droevendaalsesteeg 1, 6708 PB Wageningen, the Netherlands; Department of Microbiology and Cell Science, University of Florida, 1355 Museum Dr, Gainesville, FL 32603, United States; Department of Microbiology and Cell Science, University of Florida, 1355 Museum Dr, Gainesville, FL 32603, United States; Bioinformatics Group, Wageningen University & Research, Droevendaalsesteeg 1, 6708 PB Wageningen, the Netherlands; The Novo Nordisk Foundation Center for Biosustainability, Technical University of Denmark, 2950 Kongens Lyngby, Denmark; The Novo Nordisk Foundation Center for Biosustainability, Technical University of Denmark, 2950 Kongens Lyngby, Denmark; School of Life Sciences, University of Warwick, Coventry CV4 7AL, United Kingdom; VIB-KU Leuven Center for Microbiology, Kasteelpark Arenberg 31, 3001 Leuven, Belgium; Department of Biology, KU Leuven, Kasteelpark Arenberg 31, 3001 Leuven, Belgium; Bioinformatics Group, Wageningen University & Research, Droevendaalsesteeg 1, 6708 PB Wageningen, the Netherlands; Institute of Molecular Bio Science, Goethe-University Frankfurt, Max-von-Laue-Straße 9, 60438 Frankfurt am Main, Germany; Interfaculty Institute of Microbiology and Infection Medicine Tübingen (IMIT), Interfaculty Institute for Biomedical Informatics (IBMI), University of Tübingen, Auf der Morgenstelle 24, 72076 Tübingen, Germany; Bioinformatics Group, Wageningen University & Research, Droevendaalsesteeg 1, 6708 PB Wageningen, the Netherlands; Bioinformatics Group, Wageningen University & Research, Droevendaalsesteeg 1, 6708 PB Wageningen, the Netherlands; Bioinformatics Group, Wageningen University & Research, Droevendaalsesteeg 1, 6708 PB Wageningen, the Netherlands; Bioinformatics Group, Wageningen University & Research, Droevendaalsesteeg 1, 6708 PB Wageningen, the Netherlands; Department of Biochemistry, University of Johannesburg, C2 Lab Building 224, Kingsway Campus, Cnr University & Kingsway Road, Auckland Park, Johannesburg 2006, South Africa; Interfaculty Institute of Microbiology and Infection Medicine Tübingen (IMIT), Interfaculty Institute for Biomedical Informatics (IBMI), University of Tübingen, Auf der Morgenstelle 24, 72076 Tübingen, Germany; Institute of Molecular Bio Science, Goethe-University Frankfurt, Max-von-Laue-Straße 9, 60438 Frankfurt am Main, Germany; Senckenberg Gesellschaft für Naturforschung, Senckenberganlage 25, 60325 Frankfurt am Main, Germany; VIB-KU Leuven Center for Microbiology, Kasteelpark Arenberg 31, 3001 Leuven, Belgium; Department of Biology, KU Leuven, Kasteelpark Arenberg 31, 3001 Leuven, Belgium; School of Life Sciences, University of Warwick, Coventry CV4 7AL, United Kingdom; Department of Microbiology and Cell Science, University of Florida, 1355 Museum Dr, Gainesville, FL 32603, United States; Institute of Biology, Leiden University, Sylviusweg 72, 2333 BE Leiden, the Netherlands; Institute of Biology, Leiden University, Sylviusweg 72, 2333 BE Leiden, the Netherlands; Bioinformatics Group, Wageningen University & Research, Droevendaalsesteeg 1, 6708 PB Wageningen, the Netherlands; The Novo Nordisk Foundation Center for Biosustainability, Technical University of Denmark, 2950 Kongens Lyngby, Denmark

## Abstract

Microorganisms synthesize small bioactive compounds through their secondary or specialized metabolism. Those compounds play an important role in microbial interactions and soil health, but are also crucial for the development of pharmaceuticals or agrochemicals. Over the past decades, advancements in genome sequencing have enabled the identification of large numbers of biosynthetic gene clusters directly from microbial genomes. Since its inception in 2011, antiSMASH (https://antismash.secondarymetabolites.org/), has become the leading tool for detecting and characterizing these gene clusters in bacteria and fungi. This paper introduces version 8 of antiSMASH, which has increased the number of detectable cluster types from 81 to 101, and has improved analysis support for terpenoids and tailoring enzymes, as well as improvements in the analysis of modular enzymes like polyketide synthases and nonribosomal peptide synthetases. These modifications keep antiSMASH up-to-date with developments in the field and extend its overall predictive capabilities for natural product genome mining.

## Graphical Abstract

**Graphical Abstract:**
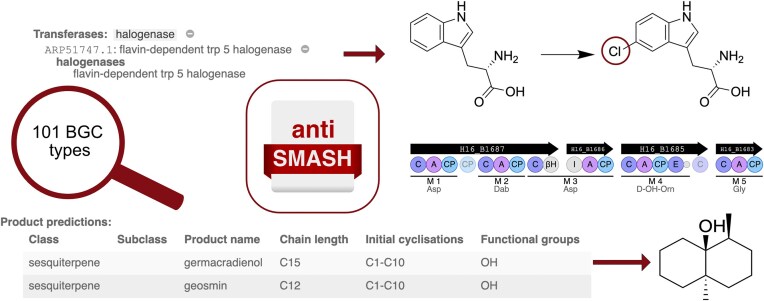

## Introduction

Small, bioactive molecules produced by microorganisms are an important source for drugs [1] and agrochemicals [2], and play important roles in microbial interactions and soil health. Over the past 20–25 years, the abundance of available genome data has allowed enhancing the traditional workflows of isolating microbial strains, extracting compounds, and then screening for desired activities by searching for biosynthetic gene clusters (BGCs) encoding for the biosynthesis of these molecules in microbial genomes [3]. Software tools for searching genomes for these secondary/specialized metabolite-producing BGCs have existed for over a decade [4–7].

Since its initial release in 2011, antiSMASH [8–14] has become the leading tool in the field. Around antiSMASH, a wide ecosystem of related tools that incorporate or rely on antiSMASH predictions has evolved. Examples include the resistance-based mining tool ARTS [15], the mass-spectrometry-guided Seq2PKS [16], the genome-engineering tool StreptoCAD [17], the BGC networking and clustering tool BiG-SCAPE [18], and the paired omics analysis tool NPLinker [19]. In turn, antiSMASH can also incorporate BGC predictions from other tools [13]. Originally built for DeepBGC [20], other machine-learning-based tools like GECCO [21] also provide their results in the required format. antiSMASH BGC predictions are also used in many genomic and BGC-related databases, such as the Joint Genome Institute’s Secondary Metabolite Collaboratory [22], the MicroScope platform for genome annotation and analysis [23], the MIBiG database of manually curated BGCs [24], the BGC family database BiG-FAM [25], the chemical diversity metagenome database BGC Atlas [26], and the antiSMASH database [27]. Furthermore, antiSMASH is part of several systematic workflows for large-scale analyses of genomic data, e.g. BGCFlow [28] or the MicroOrganisms Pipelines Service [29] of the European Food Safety Authority (EFSA).

Here, we present version 8 of antiSMASH. This release increases the number of detectable biosynthetic pathway types from 81 to 101, adds an analysis module for terpenoid BGCs, and provides in-depth analysis of tailoring enzymes. Additionally, the KnownClusterBlast and ClusterCompare datasets were updated to reflect the data from MIBiG release 4, we added proper support for BGCs spanning the origin of replication in circular genomes, and transcription factor binding site predictions were extended with datasets from the CollecTF database [30]. In BGCs containing nonribosomal peptide synthetases (NRPSs) or type I polyketide synthases (PKSs), more biosynthetic domains are detected and analyzed.

## New features and updates

### BGC detection updates

antiSMASH uses manually curated rules to define what biosynthetic functions need to exist in a genomic region in order to define a BGC. To identify these biosynthetic functions, antiSMASH makes use of both profile hidden Markov models (pHMMs) and, to a lesser extent, dynamic profiles specified in Python code files. The pHMMs are sourced from public datasets such as PFAM [31], TIGRFAMS [32], SMART [33], BAGEL [34], Yadav *et al.* [35], or created specifically for use in antiSMASH. antiSMASH 7 contained 81 of such BGC rules [14], this number has increased to 101 in this release, with a number of existing rules having been refined. Due to the large overlap in biosynthetic enzymes, it is hard to differentiate between linear azole-containing peptides and the thio-linked circularized thiopeptides. In the current antiSMASH database [27], ∼16% of thiopeptide and ∼29% of linear azole-containing peptide BGC calls overlap, and it is unclear how many of the remaining assignments are correct. To address this uncertainty and avoid potential confusion, the detection rules for both types of BGCs were merged into a new “azole-containing RiPPs rule”. The detection rules for terpenes, mycosporines, NRPS-independent siderophores, *trans*-AT PKSs, NRPSs, NRPS-like clusters, and fatty acids were also updated. Additionally, new rules for archaeal ribosomally synthesized and post-translationally modified peptides (RiPPs), atropopeptides, nitropropanoic acid, azoxy-containing compounds, polyynes, deazapurines, polyhalogenated pyrroles, hydroxytropolones, hydrogen-cyanides, darobactins, isocyanides, bacterial and fungal cyclic dipeptides, triceptides, highly reducing type II PKSs, and fungal NRPS-like lysine biosynthesis were added.

To cover cases where contiguous sections of some core genes were too far away from other core genes, a new “EXTENDS” condition was added to the rule definition to ensure that all core genes are properly marked. The *trans*-AT PKS rules use this to ensure that all genes containing modules are detected correctly, even when the single *trans*-acting acyltransferase (AT) domain is far away.

### Terpene analysis

**Figure 1.**
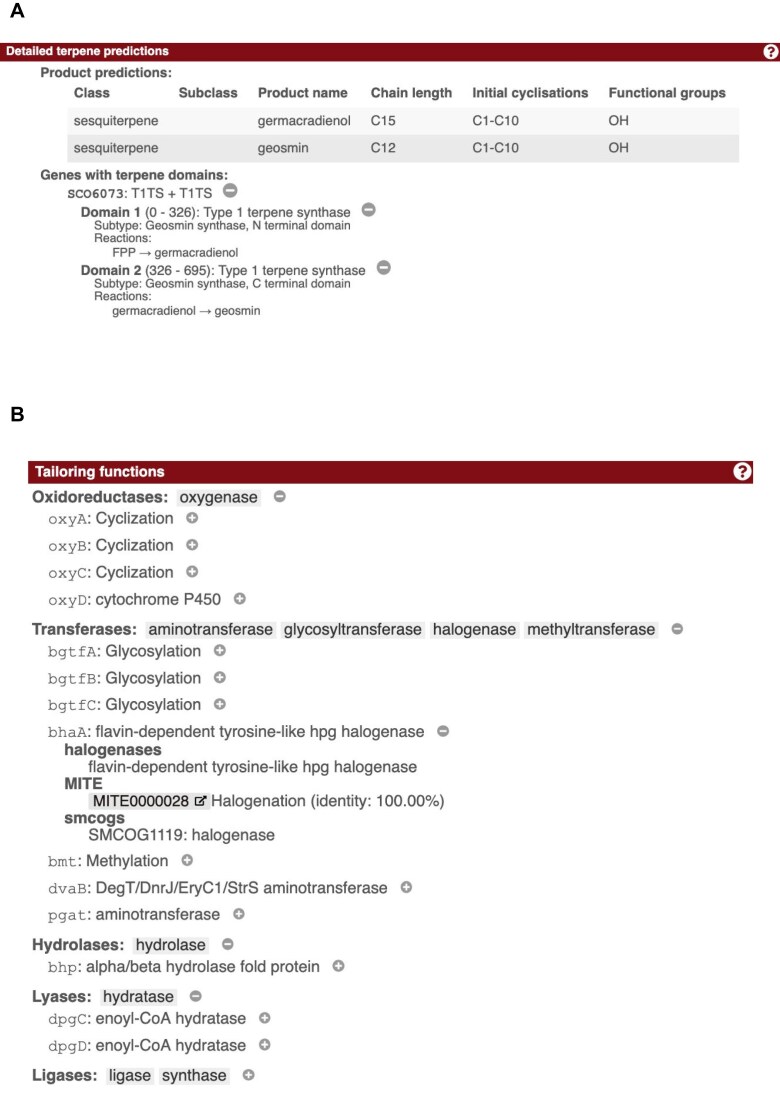
(**A**) The “terpene” tab of the geosmin BGC of *Streptomyces coelicolor* A3(2) (NCBI ID NC_003888.3:6656219–6678399) with all details expanded. The product predictions cover both the geosmin precursor germacradienol and geosmin itself. (**B**) Tailoring functions identified in the balhimycin BGC of *Amycolatopsis balhimycina* (NCBI ID Y16952.3). The oxidoreductase category has been expanded to show that all four P450 monooxygenases were identified. OxyA–C are correctly annotated as having a cyclization function due to their similarity to the MITE entries of the corresponding cyclization enzymes from the vancomycin biosynthesis. In the transferases category, the bhaA halogenase entry has been expanded further to show the predictions based on the built-in halogenase prediction, MITE similarity, and smCoG-based gene function annotations.

While antiSMASH has been detecting terpene gene clusters since version 1 [8], a more detailed analysis of the terpene synthases/cyclases was missing. antiSMASH version 4 [11] added an initial analysis module, but due to limitations in performance of the phylogenetic placement algorithm and maintainability challenges, this was dropped again in antiSMASH 5 [12]. For version 8, we see a return of the terpene analysis based on carefully curated pHMMs (see Fig. 1A). For every region containing a terpene BGC, a list of potential product types is shown. Every prediction includes the terpenoid class (e.g. diterpene, sesquiterpene, etc.) and chain length of the predicted product. For more well-understood terpene synthase subfamilies, the prediction can also contain the terpenoid subclass (e.g. indole diterpenoid), initial cyclizations (e.g. C1–C15), and product name.

### Gene function analysis

During the big community push to better annotate tailoring enzymes in MIBiG entries during last year’s MIBiG annotathons (see [24] for a description), we noticed that a more user-friendly interface to access tailoring enzyme reactions was needed in antiSMASH. Using the collection of tailoring enzymes in the MITE database (https://mite.bioinformatics.nl/) [36] annotated during those annotathons, as well as our existing smCOG annotations, we now present tailoring enzyme information in a dedicated “tailoring” tab (see Fig. 1B). This also gave us an opportunity to report the substrate specificities of many flavin-dependent halogenases, also added in this release. Using custom pHMMs and conserved motif signatures, predictions range in detail from “halogenated pyrrole” to regioselectivity of e.g. tryptophan halogenases, depending on data availability.

In the tailoring tab, tailoring enzymes are organized by Enzyme Commission category, i.e. oxidoreductases, transferases, hydrolases, lyases, isomerases, and ligases. Only categories with hits in the region are shown. Clicking the plus icons expands the category to show the genes with relevant hits and a short summary of the most detailed prediction of the tailoring function possible. Clicking on the plus icon of a gene of interest expands all information antiSMASH provides about a tailoring enzyme. If a tailoring enzyme shows at least 60% amino acid sequence identity to any entry in the MITE database, a cross-link to that MITE entry is provided.

### NRPS and PKS improvements

antiSMASH provides a detailed analysis of protein domains encoded in NRPS/PKS BGCs. To provide a more comprehensive overview of the domains present, we added profiles for siderophore-associated β-hydroxylases and interface domains (see [37] for a detailed discussion) and a more generic α/β-hydrolase profile, as some enzymes with presumed proofreading functions were missed by the existing specific thioesterase profile.

CoA-ligase (CAL) domains are often involved in loading fatty acid-derived starter units in lipopeptide BGCs, but were not previously considered starting modules in the antiSMASH module detection. Together with the aforementioned β-hydroxylases and interface domains they can now be detected as part of modules.

Following the information collected in [38], the active sites of NRPS condensation (C) and epimerization (E) domains are now checked for the presence of catalytic residues and flagged as inactive when those residues are missing. To complement the NRPS adenylation (A) domain substrate specificity predictions already performed in antiSMASH, we now also provide a link to the external PARAS substrate specificity predictor [39] to provide researchers with even more analysis options.

### Miscellaneous changes

Many other smaller changes have been included in version 8. The overview page view showing the most similar known clusters has been simplified to address some sources of user confusion. Instead of directly showing the cluster similarity in “percent of the genes having a sequence similarity of at least 30%”, we now show the similarity in three confidence levels: “high” for a cluster similarity of larger or equal to 75%, “medium” for a cluster similarity between 75% and 50%, and “low” for a cluster similarity between 50% and 15%. Cluster similarities of <15% are no longer considered to be similar enough and are no longer shown in the overview. KnownClusterBlast, ClusterCompare, and CompaRiPPson have been updated to the data provided in the MIBiG 4.0 release [24]. ClusterBlast SVG generation was moved into JavaScript to reduce file sizes and improve the user experience of viewing antiSMASH results opened locally. Historically, antiSMASH has struggled with BGCs spanning the origin in circular genomes. At best, those BGCs were split in two, at worst one or both of the parts on the opposite sides of the origin could be missed entirely. In antiSMASH 8.0, we have completely overhauled our coordinate handling for circular genomes to be detected and reported properly.

Historically, antiSMASH has relied on GlimmerHMM [40] for fungal gene calling, as it was able to run without manually selecting the right gene model. Unfortunately, with more and more fungal genomes becoming available, it became evident that this gene model selection is crucial for high-quality gene calling. As we cannot easily select the right gene model for uploaded genomes of unknown taxa, we have decided to remove fungal gene calling functionality from antiSMASH. We recommend users to run a dedicated gene-calling tool like AUGUSTUS [41] and then provide antiSMASH with the gene annotations. Due to software incompatibilities in modern systems, we have also deprecated our support for the MEME suite of tools, specifically MEME [42] and FIMO [43]. Users of the standalone version of antiSMASH can still provide those binaries themselves, but they are no longer part of the containers we provide or the web service. This effectively disables the CASSIS [44] fungal BGC border detection and might affect the RODEO [45] score for lanthipeptide, sactipeptide, lasso peptide, and azole-containing RiPP precursors.

## Conclusion and future perspective

Genome mining technologies like antiSMASH constitute an important piece of the natural product discovery puzzle. Over the past 14 years, antiSMASH has seen continuous updates and improvements, making sure it stays at the forefront of natural product genome mining tools. As evident by the numerous contributions from outside of the core development team, the antiSMASH project’s Open Source and Open Science model remains successful. It is an important tool that many other, more specialized, tools, workflows, and databases rely on. It serves as the technology platform for a number of other genome mining tools currently in development. In future updates, we will continue our work on new algorithms and analysis modules to decipher the full biosynthesis pathways of detected clusters. We will build on the foundation of the tailoring enzyme prediction improvements in this version and cover even more enzyme families. Similarly, our work on regulator and regulator binding site detection will continue. As always, we will also integrate, or integrate with, new tools developing in the ecosystem.

## Data Availability

The bacterial and fungal versions of antiSMASH 8.0 can be freely accessed at https://antismash.secondarymetabolites.org and https://fungismash.secondarymetabolites.org, respectively. The antiSMASH documentation is available at https://docs.antismash.secondarymetabolites.org/. The antiSMASH source code is licensed under the GNU Affero General Public License (AGPL) version 3.0. antiSMASH is also available via Docker. See the documentation website for details on how to download and install antiSMASH.
